# A progesterone-brown fat axis is involved in regulating fetal growth

**DOI:** 10.1038/s41598-017-10979-7

**Published:** 2017-09-06

**Authors:** Saraid McIlvride, Aleena Mushtaq, Georgia Papacleovoulou, Chloe Hurling, Jennifer Steel, Eugène Jansen, Shadi Abu-Hayyeh, Catherine Williamson

**Affiliations:** 1 Women’s Health Academic Centre, King’s College London, London, SE1 1UL United Kingdom; 2 Institute of Reproductive and Developmental Biology, Imperial College London, London, W12 0NN United Kingdom; 3 Centre for Health Protection, National Institute for Public Health and the Environment, 3720 BA Bilthoven, The Netherlands

## Abstract

Pregnancy is associated with profound maternal metabolic changes, necessary for the growth and development of the fetus, mediated by reproductive signals acting on metabolic organs. However, the role of brown adipose tissue (BAT) in regulating gestational metabolism is unknown. We show that BAT phenotype is lost in murine pregnancy, while there is a gain of white adipose tissue (WAT)-like features. This is characterised by reduced thermogenic capacity and mitochondrial content, accompanied by increased levels of markers of WAT and lipid accumulation. Surgical ablation of BAT prior to conception caused maternal and fetal hyperlipidemia, and consequently larger fetuses. We show that BAT phenotype is altered from day 5 of gestation, implicating early pregnancy factors, which was confirmed by reduced expression of BAT markers in progesterone challenged oophorectomised mice. Moreover, *in vitro* data using primary BAT cultures show a direct impact of progesterone on expression of *Ucp1*. These data demonstrate that progesterone mediates a phenotypic change in BAT, which contributes to the gestational metabolic environment, and thus overall fetal size.

## Introduction

Pregnancy is associated with a gradual metabolic shift from normal maternal serum concentrations of triglycerides, free fatty acids, and glucose at the time of conception to hyperlipidemia and insulin resistance in the third trimester. These changes support the growth and development of the fetus. The importance of maternal-fetal energy transfer is exemplified by several pathophysiological conditions. Under-nutrition or obesity during pregnancy can increase the risk of babies being born with intrauterine growth restriction or large for gestational age^1–4^. Furthermore, altered metabolism as a result of maternal obesity is associated with an increased risk of gestational diabetes and pre-eclampsia^5–7^, and predisposes the infant to metabolic disease in later life^8–10^.

Maternal lipid homeostasis is dependent upon the coordination of metabolically active organs. For example, during early pregnancy, increased insulin sensitivity in white adipose tissue (WAT) results in lipid accumulation, whereas towards term, in conjunction with placental hormones, there is insulin resistance and lipid catabolism^11^. However, little is known about the prevalence and function of brown adipose tissue (BAT) during pregnancy. Adult humans possess classical brown adipocytes and beige cells of white adipocyte origin, both of which are thermogenic and can contribute to whole body energy metabolism (reviewed in ref. 12). Interestingly, women have been found to possess larger volumes of BAT than men^13,14^. It is not known whether BAT is involved in regulating the gestational metabolic profile. Nonetheless it has been hypothesised that there is a decrease in BAT-mediated energy expenditure during pregnancy to divert energy to the developing fetus and/or lactation (reviewed in refs 15 and 16), although the data to support this are inconsistent. Research into the effects of reproductive hormones on BAT in the context of pregnancy has largely focused on estrogen^17, 18^ with limited studies of progesterone and prolactin^19, 20^.

In this report, we examine the impact of pregnancy on BAT, the role of BAT in regulating the maternal and fetal metabolic environment, as well as alterations in BAT phenotype mediated by early pregnancy signals.

## Results

### BAT undergoes morphometric and functional changes during pregnancy

**Figure 1:**
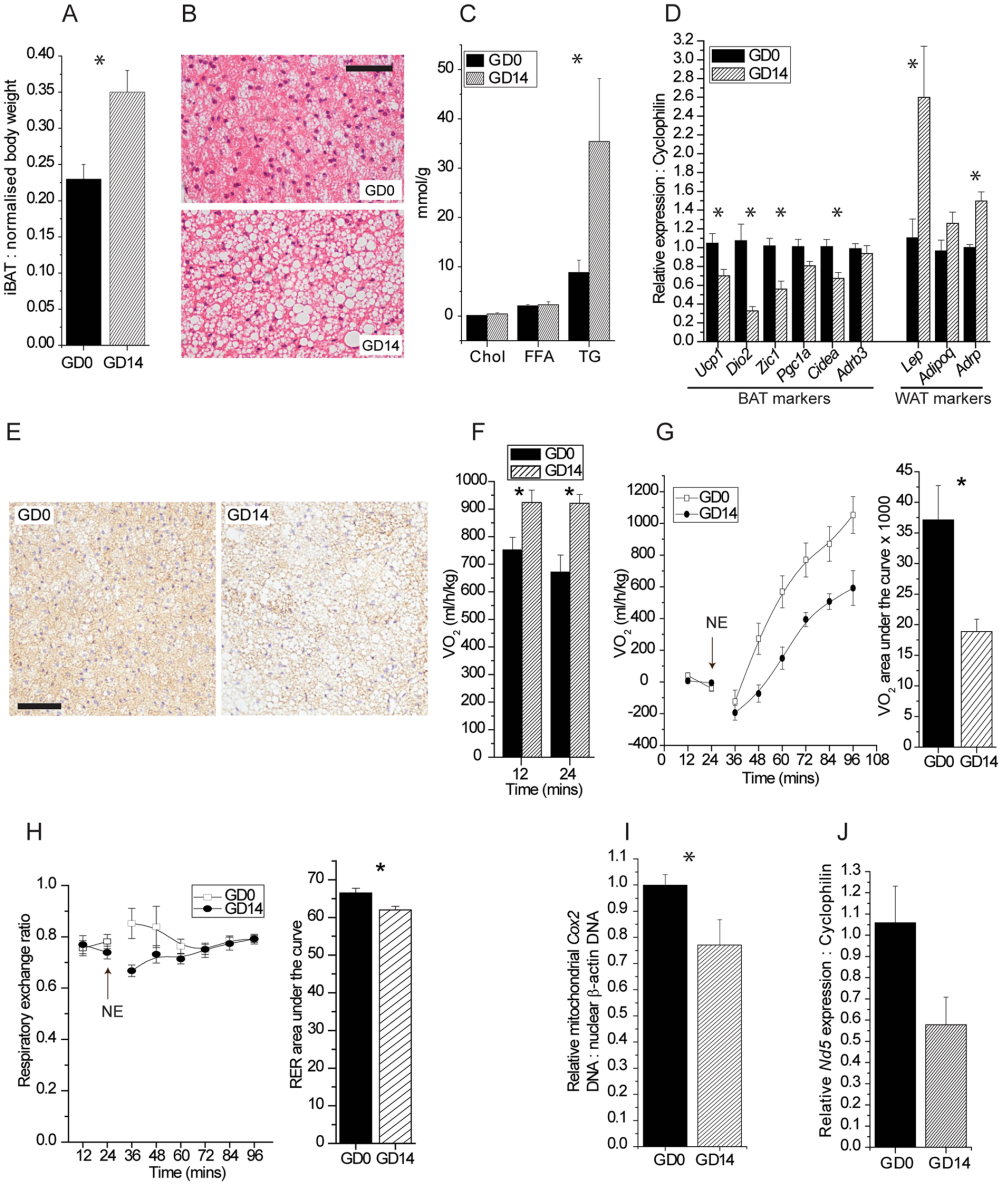
Pregnancy mediates changes in the morphometry and function of BAT. (**A**) Interscapular BAT (iBAT) weight expressed as a percent of total body weight without uterine horn at gestational days 0 (GD0) and 14 (GD14). (**B**) H&E staining of iBAT. Scale bar, 100 µm. (**C**) Concentrations of total cholesterol (Chol), free fatty acids (FFA) and triglycerides (TG), normalised to protein, in iBAT. (**D**) Expression of genes related to BAT and WAT phenotype. (**E**) Immunohistochemical staining for UCP1 in iBAT. Scale bar, 100 µm. (**F**) Basal measurement of O_2_ consumption in terminally anaesthetised mice. (**G**) Measurement of norepinephrine (NE)-mediated O_2_ consumption in terminally anaesthetised mice over time with O_2_ consumption total area under the curve. To facilitate comparisons, responses are expressed as increases over pre-NE values. (**H**) NE-mediated changes in respiratory exchange ratio (RER) over time with RER total area under the curve. (**I**) qPCR analysis of DNA levels of mitochondrial *Cox2* normalised to nuclear β-actin in iBAT. (**J**) Expression of the mitochondrial-encoded gene *Nd5* in iBAT.

To understand the impact of brown adipose tissue (BAT) on pregnancy, a period associated with profound metabolic change, we first identified a point during gestation to study BAT biology that follows significant alterations in serum lipid concentrations. Longitudinal sampling of sera over the course of pregnancy identified gestational day (GD) 14 as a point that follows significant increases in serum cholesterol, HDL, and triglyceride concentrations (see Supplementary Fig. S1); fasting serum glucose concentrations were not different at this time point (data not shown). Interscapular BAT (iBAT) normalised against total body weight without the uterine horn was found to be significantly heavier by 52% at GD14 relative to non-pregnant controls (GD0) (Fig. 1A, and Supplementary Figure S2). H&E staining and lipid biochemical analyses revealed that the iBAT at GD14 contained enlarged lipid droplets (Fig. 1B), and that there was a 4-fold increase in triglyceride (TG) concentrations, respectively (Fig. 1C). iBAT cholesterol and free fatty acids (FFA) concentrations were unchanged. Profiling of classical phenotypic markers of brown and white adipose tissue revealed significantly decreased relative gene expression of BAT markers *Ucp1*, *Dio2*, *Zic1*, and *Cidea*, accompanied by significant increases in white adipose tissue (WAT) markers *Lep* and *Adrp*. Gene expression of the β3-adrenergic receptor was unchanged (Fig. 1D). Immunohistochemical analysis of GD14 BAT revealed decreased levels of UCP1 (Fig. 1E). Cumulatively, these data point to a decrease in overall BAT phenotype accompanied by ‘whitening’ of the tissue at GD14.

To test whether the reduction of BAT phenotype translated to a decrease in basal and inducible thermogenic BAT function, indirect calorimetry was used to measure O_2_ consumption in terminally anaesthetised mice at GD14. Basal O_2_ consumption was found to be significantly higher than at GD0 (Fig. 1F). However, pregnancy at GD14 significantly blunted the acute response to the β3-adrenergic receptor agonist norepinephrine (NE), as inducible O_2_ consumption was reduced by 49% (Fig. 1G) and the respiratory exchange ratio by 7.3% (Fig. 1H). Relative iBAT mitochondrial *Cox2* DNA levels were significantly decreased at GD14, and mRNA expression of the mitochondrial gene *Nd5* was also markedly reduced, albeit not significantly (Fig. 1I,J). Taken together with the UCP1 expression result, these data indicate reduced BAT thermogenic capacity.

### Ablation of maternal BAT perturbs gestational metabolism and fetal growth

**Figure 2:**
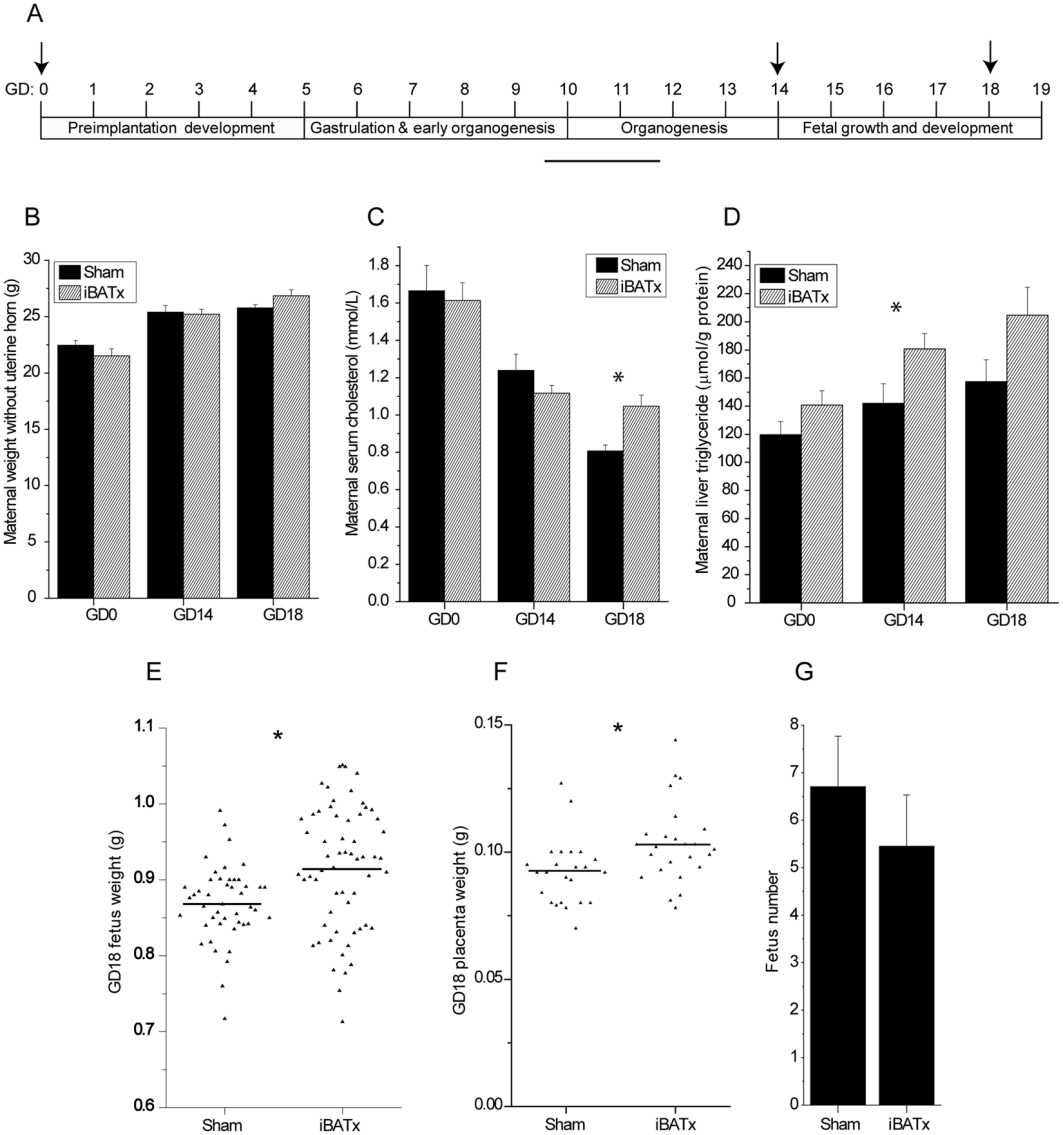
Surgical ablation of interscapular BAT alters maternal and fetal metabolic profiles. (**A**) Schema summarising the key stages of mouse pregnancy over time and the time-points selected for the study of pregnancy and metabolism in iBAT ablated (iBATx) or sham-operated mice, as indicated by the arrows. (**B**) Maternal weight without uterine horn (Sham, n = 5–10; iBATx, n = 6–9). (**C**) Maternal serum cholesterol levels (Sham, n = 6–9; iBATx, n = 7–10). (**D**) Maternal liver triglyceride levels (Sham, n = 7–10; iBATx, n = 9–12). Data are represented as mean ± SEM, *p<0.05 for Sham versus iBATx comparison at a single pregnancy time-point as determined by unpaired Student’s t-test. (**E**) Weight of fetuses at GD18 (Sham, n = 46 fetuses from 7 mothers; iBATx, n = 51 fetuses from 9 mothers). (**F**) Weight of placentas at GD18 (Sham, n = 24 placentas from 4 mothers; iBATx, n = 25 placentas from 5 mothers). (**G**) Average number of fetuses per pregnancy at GD18 (Sham n = 7; iBATx n = 9). *p<0.05 as determined by unpaired Student’s t-test.

We hypothesised that the gestational loss of classical BAT phenotype and function with accompanied whitening is involved in supporting the maternal and fetal metabolic environment. A surgical approach was employed to ablate the interscapular BAT depot (iBATx) of female mice prior to conception, to enable assessment of the relative contribution of BAT to gestational metabolism. Gestational days 14 and 18 were chosen as time points of interest to capture the impact of BAT ablation prior to, and at the end of, the period of rapid fetal growth and development (Fig. 2A). Ablation of BAT had no effect on maternal weight; however, serum cholesterol levels were significantly increased by 29.7% at GD18 (Fig. 2B,C). Serum triglycerides, FFA and fasted glucose levels remained unchanged (see Supplementary Fig. S3). In the BAT-ablated group, maternal hepatic triglycerides were increased by 17.6%, 27.2% and 30.0% at GD0, GD14 and GD18, respectively, although the change was only significant for GD14 (Fig. 2D). No differences were observed in liver cholesterol and FFA levels (see Supplementary Fig. S3). Importantly, there was no evidence of compensatory ‘browning’ of WAT in the iBATx mice (see Supplementary Fig. S5).

The impact of BAT ablation on fetal parameters was evident at GD18, as the average fetal and placental weight increased by 7.8% and 12.3% respectively (Fig. 2E,F), whereas at GD14 no differences were observed (see Supplementary Fig. S4). Therefore, at a macroscopic level, fetal growth is influenced by maternal BAT. There was no significant difference in the average number of fetuses per pregnancy between the two groups at GD18 indicating that the increase in fetal and placental weight in the iBATx group is due to the decrease of a factor or function associated with brown fat during pregnancy (Fig. 2G).

### Ablation of maternal BAT perturbs maternal and fetal lipid homeostasis

**Figure 3:**
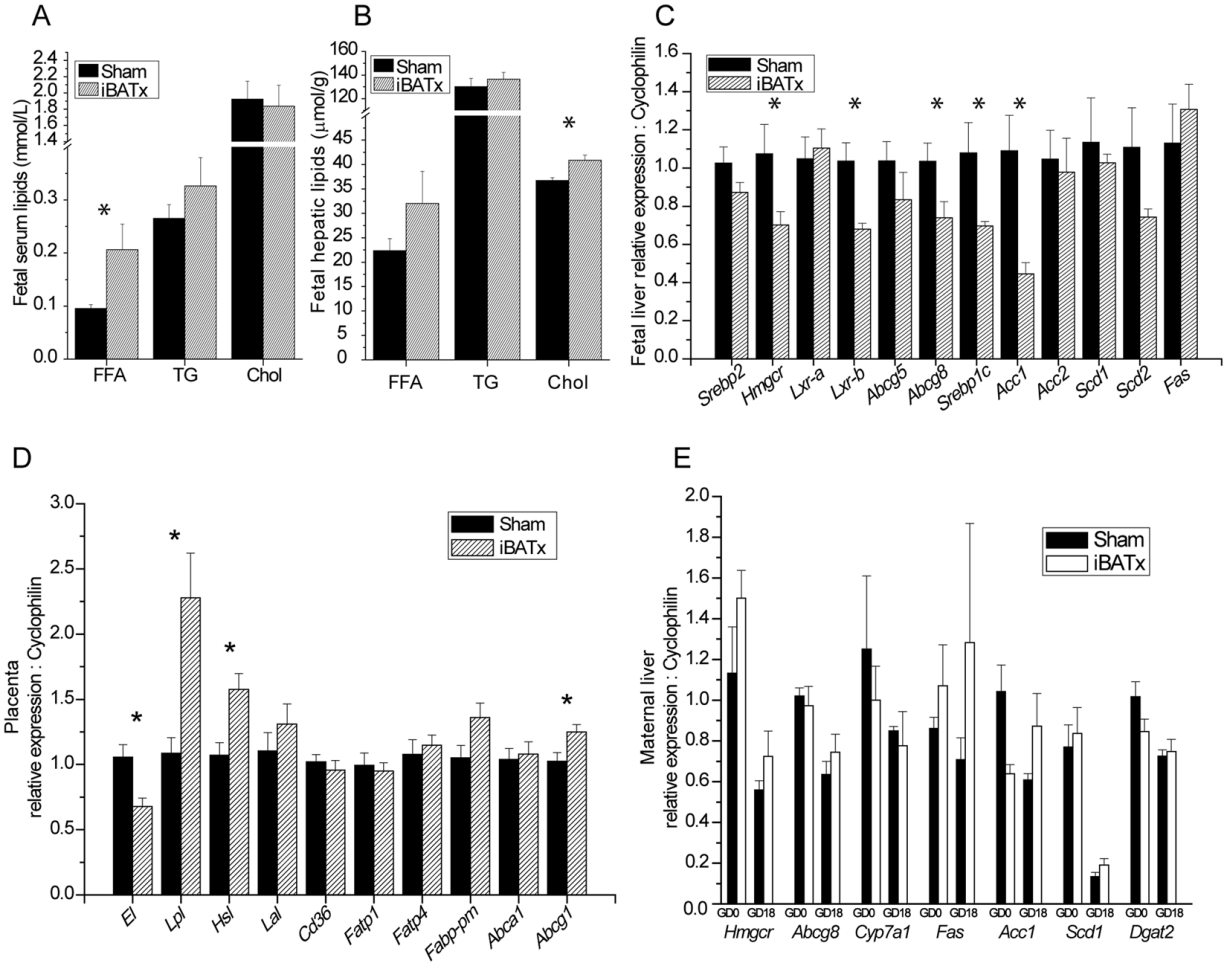
Surgical ablation of maternal interscapular BAT results in fetal hyperlipidemia. Serum concentrations (**A**) and normalised hepatic levels (**B**) of total free fatty acids (FFA), triglycerides (TG) and total cholesterol of GD18 fetuses from sham-operated (n = 4) or iBATx (n = 5–6) mothers. Expression of genes related to lipid homeostasis in (**C**) GD18 fetal liver (n = 4 per group), (**D**) GD18 placenta (Sham, n = 7; iBATx, n = 10)) and (**E**) GD0 and 18 maternal liver (Sham, n = 6–7; iBATx, n = 7–8). Liver and serum were pooled from fetuses from the same mother. Data are represented as mean ± SEM, *p<0.05 as determined by unpaired Student’s t-test.

The mouse fetus undergoes a rapid growth phase between GD14 and parturition, which coincides with our observation that at GD18, the fetuses are heavier from iBATx mothers. This may be as a result of altered fetal or maternal lipid homeostasis and fuel availability. Biochemical analysis of FFA concentrations in the serum of fetuses at GD18 from iBATx mothers revealed a significant increase of 117% (Fig. 3A), whereas triglyceride and total cholesterol levels remained unchanged. GD18 fetal hepatic cholesterol concentrations were significantly increased by 11.4% and there was a trend for increased FFA in the iBATx group (Fig. 3B).

To determine the source of the increased FFA levels, the expression of genes involved in lipid metabolism was studied in GD18 fetal and maternal liver as well as placenta. Fatty acid gene expression targets were either unchanged or significantly decreased, e.g. *Srebp1c* and *Acc1* in the fetal liver (Fig. 3C). The expression of placental lipases was studied, as they may be involved in the hydrolysis of serum triglycerides to fatty acids, which are transported across into the fetoplacental unit. Indeed, the expression of *Lpl* and *Hsl* were significantly increased by 2.3- and 1.6-fold respectively (Fig. 3D). There was a trend for increased gene expression of maternal hepatic enzymes *Acc1* and *Scd1* involved in generating FA in iBATx mothers (Fig. 3E). These data suggest that the increased FFA in the fetuses from the iBATx mice is not due to *de novo* lipogenesis, but rather increased transport across the placenta as indicated by the upregulation of placental lipolysis.

### Early gestational alterations in BAT phenotype are mediated by a progesterone signal

**Figure 4:**
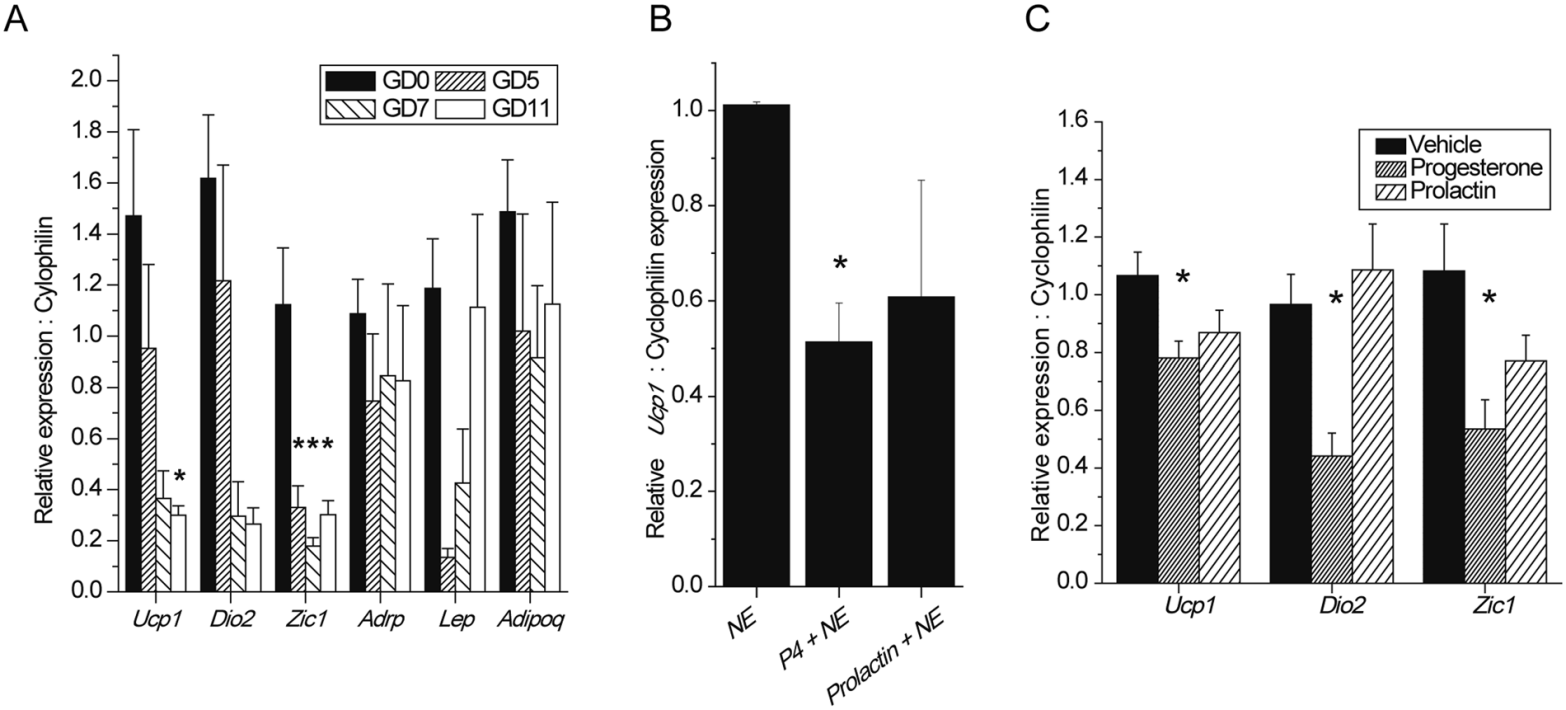
Progesterone mediates a reduction in the expression of thermogenic genes in brown adipose tissue. (**A**) Expression of genes related to BAT and WAT in iBAT at GD0 (n = 4), GD5 (n = 5), GD7 (n = 5) and GD11 (n = 5). (**B**) Relative *Ucp1* expression of cultured BAT following treatment with 1 μM norepinephrine (NE) ± 500 nM progesterone (P4) or 550 ng/ml prolactin normalised to the 1 μM norepinephrine treated group (n = 4). (**C**) Normalised relative gene expression of BAT targets in oophorectomised mice challenged with vehicle (n = 7), progesterone (n = 6) or prolactin (n = 6) for 14 days via osmotic pumps. All data are represented as mean ± SEM. *p<0.05 as determined by one-way ANOVA.

To delineate the gestational signal that alters the BAT phenotype, the expression profiles of BAT and WAT markers were studied in the iBAT from pregnant mice at GD5 and GD7, pre-placentation time points, and GD11, post-placentation. *Ucp1* and *Dio2* gene expression was reduced considerably from as early as GD7, and *Zic1* was significantly reduced from as early as GD5, indicating that pre-placentation factors are involved in mediating gestational gene expression changes in BAT. The gene expression of the WAT marker leptin was markedly decreased at GD5 and GD7, returning to control levels by GD11. The gene expression of the WAT marker adiponectin did not change at the time-points studied (Fig. 4A). These data are supported by the fact that BAT weight is unchanged at GD5 (see Supplementary Fig. S6), therefore lipid accumulation does not occur until the later stages of pregnancy. To identify the pre-placentation hormone that is able to mediate the early gestational changes in BAT phenotype, an *in vitro* approach was taken. Differentiated primary brown adipocytes were treated with 500 nM progesterone or 550 ng/ml prolactin overnight, which are physiological concentrations^10, 21^, ± a 2 hour treatment with 1 μM of the β3-adrenergic agonist NE. *Ucp1* gene expression was used as a quantifiable reporter due to it being a functional marker of brown adipose tissue and because its induction is measurable following a 2-hour acute challenge. Progesterone significantly inhibited NE-mediated induction of *Ucp1* by 49%, whereas prolactin did not exert any significant effects (Fig. 4B). This result demonstrated that the pre-placentation hormone progesterone can blunt the induction of the BAT marker *Ucp1*. Finally, we sought to recapitulate the *in vitro* data in an *in vivo* model. Exogenous progesterone was administered chronically over two weeks by an osmotic pump to oophorectomised mice, which resulted in a significant relative reduction in the expression of *Ucp1*, *Dio2* and *Zic1*. There was a trend for prolactin to inhibit *Ucp1* and *Zic1* expression, but not to the extent of progesterone (Fig. 4C), the concentration of which rises early in pregnancy (see Supplementary Fig. S7). Concentrations of cholesterol, triglycerides, FFA and glucose, as well as mouse weight were unchanged (data not shown).

These data are consistent with progesterone being the endocrine signal that mediates the gestational changes in BAT phenotype.

## Discussion

Fluxes in energy metabolism during pregnancy are vital for the controlled growth of the developing fetus. We have demonstrated throughout this study the importance of pregnancy-mediated adaptations to BAT, which contribute to the regulation of energy metabolism in both the mother and the fetus. We conclusively show at a macroscopic level that the iBAT was hypertrophied at GD14 of pregnancy due to an increase in triglyceride concentrations and lipid droplet size. This was accompanied by a decrease in the levels of markers of classical BAT phenotype and a parallel increase in WAT markers, giving the tissue a whitened phenotype.

To ascertain if the diminished BAT phenotype resulted in decreased BAT function, we assessed the capacity of β3-adrenergic agonist-stimulated thermogenesis at GD14 using indirect calorimetry, avoiding the muscular contribution to O_2_ consumption by terminally anaesthetising the mice. As previously reported^22^, we observed that basal O_2_ consumption was greater during pregnancy, which may be a consequence of increased metabolic activity associated with maintaining pregnancy. However, we demonstrated that norepinephrine-mediated O_2_ consumption was blunted at GD14, despite the higher basal O_2_ consumption. This was paralleled by a reduction in the norepinephrine (NE)-mediated RER value. The levels of mitochondrial markers were also reduced at GD14 and, when combined with reduced UCP1 expression and increased lipid concentrations, could be responsible for the abrogation of BAT function during pregnancy. These results demonstrate that BAT undergoes adaptive changes characterised by a loss of classical thermogenic phenotype and function accompanied by whitening during pregnancy. Similarly, whitening of BAT with a loss of BAT phenotype and function has been observed in diet-induced obese mice^23^.

Evidence for a functional role of gestationally modified BAT was provided by experiments where iBAT, which represents approximately 60% of total BAT^24^, was surgically ablated prior to conception. Days 14 and 18 of gestation were chosen to study the impact of BAT ablation, as they lie before and towards the end of the period of rapid fetal growth^25, 26^. Removal of BAT had no effect on maternal body weight. This is not surprising, as mice lacking UCP1 housed below thermoneutrality do not become obese and O_2_ consumption remains unchanged^27–29^. However, the iBATx group had significantly higher levels of serum cholesterol and hepatic triglyceride concentrations. This is consistent with the view that gestationally adapted BAT contributes to whole body lipid metabolism during pregnancy.

It is well established that the metabolic status of the mother can affect the developing fetus^30–33^. We therefore studied the consequences of iBAT ablation on the fetal compartment. Fetal and placental weight were increased at GD18 in the iBATx group as a result of a decrease of a factor or function associated with brown fat during pregnancy that contributes to the regulation of fetal growth. There are striking similarities between maternal obesity, which is associated with hypertriglyceridemia and an increased risk for babies to be born large-for-gestational age^4, 7, 31, 34, 35^, and our findings in iBAT ablated mice that have raised maternal hepatic triglyceride levels and heavier GD18 fetuses. This emphasises the importance of BAT in regulating systemic maternal lipid levels during a critical period of fetal development.

Biochemical analyses of the serum from the iBATx GD18 fetuses revealed elevated FFA which is likely responsible for the increased placental and fetal weight. Indeed, FFA are readily converted to precursors of other molecules that modulate cell growth and metabolism, and incorporated into cellular structures^36^. We hypothesised that the increased FFA levels originated from the maternal serum, as gene expression analysis of lipid homeostasis targets in the fetal liver were not increased and, in some cases, were reduced, possibly as a measure to rebalance the elevated fetal serum FFA levels. The probable source of the increased fetal serum FFA is hydrolysed triglycerides from the placenta, as the expression of placental lipases was significantly increased in GD18 iBATx mice. This may be a homeostatic response to reduce the elevated levels of GD14 maternal triglycerides (Fig. 2D), emphasising the sensitive nature of the maternal-fetal lipid metabolic axis and the important role BAT plays in this. iBATx fetal liver cholesterol concentrations were also significantly increased at GD18, reflecting the GD18 iBATx maternal hypercholesterolemic environment and increased levels of the placental cholesterol transporter *Abcg1*. However, GD18 iBATx fetal liver gene expression targets involved in cholesterol synthesis were decreased, indicating a homeostatic response to the aberrant hepatic cholesterol concentrations. The data presented here show a direct link between maternal BAT activity and fetal energy metabolism and growth. A recent study in humans has proposed that there is a crosstalk between maternal BAT and fetal adiposity^37^. In light of our study, the impact of maternal BAT ablation upon offspring metabolism in the long term warrants further investigation, particularly since adipogenesis is a potential target for fetal programming^38^.

Pregnancy factors, including reproductive hormones, alter a range of physiological systems to adapt them to the gestational needs of the mother and fetus. The impact of pregnancy hormones on BAT has been studied extensively, but the data are conflicting. Estrogen has been shown to both increase and decrease BAT activity^17, 18, 39–41^. This may be a reflection of species and strain specific differences as well estrogen delivery strategies used. In addition, previous studies of progesterone and prolactin effects indicate that both hormones can influence BAT phenotype and activity^19, 42–47^. In this study, we investigated three different model systems to identify an early gestational signal capable of eliciting phenotypic changes in the BAT seen during pregnancy. Firstly, by studying the temporal expression of BAT markers at different stages of pregnancy, we were able to define GD5 and GD7 as time-points where the expression of classical BAT markers *Ucp1*, *Dio2* and *Zic1* were reduced. At GD5 and GD7, the gestational factors are likely to be pre-placentation hormones secreted by the ovary and pituitary gland such as progesterone and prolactin respectively, whereas post-placentation, GD10 onwards, would include hormones secreted from the placenta, such as estrogen. The expression of the WAT markers leptin and adiponectin were not increased, revealing that gestational whitening of BAT, as observed at GD14, follows the reduction in the expression of classical BAT markers. Differentiated primary BAT cultures were treated with physiological concentrations of progesterone or prolactin in combination with the β3-adrenergic agonist NE. NE-mediated *Ucp1* induction was consistently blunted by the progesterone treatment, demonstrating that progesterone is a pregnancy signaling molecule that can act on brown adipocytes to modulate the expression of thermogenic targets. This result is also strongly indicative of a direct effect of progesterone on the brown adipocyte. Finally, the expression of *Ucp1*, *Dio2* and *Zic1* were all reduced by exogenous progesterone delivered by osmotic pumps in oophorectomised mice reconfirming that progesterone is a key gestational signal involved in altering BAT phenotype. This is the first study to show that the reproductive hormone progesterone consistently decreases BAT phenotype and is therefore a key mediator of the gestational metabolic switch. To corroborate our findings, experiments would be required to show that maternal BAT does not change in the absence of progesterone. However, due to the physiological importance of progesterone in maintaining pregnancy, these studies are not feasible. Further work is needed to investigate whether our findings have implications for use of progesterone in *in vitro* fertilisation techniques, or for women taking progesterone supplements as hormonal contraception or for the prevention for pre-term birth.

Human studies have shown that women possess greater volumes of BAT relative to men, which decrease with age^14^, yet few studies have sought to understand why this may be. The data presented here demonstrate that the early gestational factor progesterone switches the phenotype of brown adipose tissue, which is necessary to support the maternal and fetal metabolic milieu. Moreover, this is the first study to our knowledge that demonstrates a direct relationship between maternal BAT activity and fetal growth. These data support the notion that BAT has an underappreciated gender-specific role in women that has evolved to modify lipid metabolism during pregnancy, and underscores BAT as a potential therapeutic target of maternal obesity and as a consequence, fetal macrosomia.

## Methods

### Animal studies

Animal studies were performed using C57BL/6 mice from Charles River UK Ltd., maintained on a standard breeding diet (RM3) and allowed free access to food and water. They were housed in stable conditions (12 hour light/dark cycle; 22–24 °C). Mice were acclimatised to the animal house for 1 week prior to experiments.

For pregnancy experiments, 7–8 week old female mice were mated with males. Virgin mice were used as controls (gestational day (GD) 0). GD1 was considered to be the day the copulatory plug was identified. For all animal studies, mice were sacrificed after a 4 hour fast, and maternal and fetal serum and tissues were collected and snap-frozen immediately or fixed in 10% neutral buffered formalin for 24 hours.

### Metabolic studies

Indirect calorimetry was carried out using the LabMaster cage system (TSE). GD0 and GD14 mice were terminally anaesthetised by intraperitoneal injection of 90 mg/kg pentobarbital (Merial Animal Health Limited). Once fully sedated, they were placed in the metabolic cages and allowed to acclimatise for 20 minutes, to establish a constant metabolic baseline. Norepinephrine (NE; 1 mg/kg; Sigma-Aldrich) was then administered by subcutaneous injection at the nape. The O_2_ consumption and CO_2_ production was measured in each cage sequentially every 12 minutes, and retrospectively corrected for body weight without pups. Respiratory exchange ratio was calculated according to manufacturer’s software.

### BAT ablation surgery

7–8 week old female mice were anaesthetised via isoflurane vapour (Abbott Animal Health) and the interscapular BAT (iBAT) was surgically removed. Sham-operated mice underwent the same procedure without the removal of iBAT. Mice were single-housed for 2 weeks during the recovery period, and then paired with males until visual confirmation of a plug, following which they were monitored until sacrifice at GD14 or GD18 of pregnancy, along with GD0 controls.

### Osmotic pump implant surgery

7–8 week old female mice were anaesthetised via isoflurane vapour and underwent bilateral oophorectomy. After a two-week recovery period, the mice were anaesthetised and 14-day release osmotic minipumps (Alzet model 2002; Charles River) were implanted subcutaneously in the dorsal region. Osmotic pumps contained progesterone (250 µg/day; Sigma-Aldrich), prolactin (7 µg/day; PeproTech) or vehicle only (20% (w/v) 2-hydroxypropyl-beta-cyclodextrin, Sigma Aldrich). After 2 weeks, animals were sacrificed as described above.

### Histology and immunohistochemistry

Fixed iBAT sections were embedded in paraffin and sectioned at 5 µm onto poly-L-lysine coated slides. Sections were stained with haematoxylin and eosin for histological analysis. Immunohistochemical analysis was performed as previously described^48^. Sections were incubated overnight at 4 °C with rabbit anti-mouse primary antibody to UCP1 diluted 1:10,000 in phosphate buffered saline. The UCP1 antibody was a kind gift from Barbara Cannon.

### Lipid measurements

Serum and tissue cholesterol, LDL-cholesterol, HDL-cholesterol, TG, FFA and total proteins were measured using an Unicel DxC 800 autoanalyzer (Beckman-Coulter) using dedicated kits from Beckman-Coulter, excluding FFA which were measured using a kit from Wako Diagnostics (Neuss). Liver and brown adipose tissue were extracted in a 0.125 M potassium phosphate buffer and normalised to the total protein content.

### Primary adipocyte culture and differentiation

iBAT was removed from 10 day old C57BL/6 pups and pooled. Primary adipocytes were isolated and differentiated as previously described^49^. Cells were seeded and grown in growth media consisting of DMEM/10% NCS with (final concentrations) 20 nM insulin (Actrapid, Novonordisk) and 1nM triiodothyronine (Sigma-Aldrich), until 70% confluent. The adipocytes were differentiated for 48 hours in the above media supplemented with 500 µM isobutylmethylxanthine, 2 µM dexamethasone, 50 µM indomethacin and 1 µM rosiglitazone (Sigma-Aldrich). On the morning of an experiment the media was replaced with growth media.

### mRNA analysis

Total RNA was extracted from frozen tissue using the RNeasy Mini Kit (Qiagen), and cDNA synthesised using the Superscript II kit (Thermo Fisher Scientific), according to manufacturer’s instructions. Semi-quantitative real-time PCR with SYBR Green JumpStart Taq ReadyMix (Sigma-Aldrich) was used to detect and amplify target cDNA. Relative gene expression was calculated using the ∆∆ Ct method. Genes of interest were normalised to the housekeeping gene Cyclophilin B. See Supplementary Experimental Procedures online for a table of primers and sequences. Basal expression of *Ucp1* was undetectable in several of the cultures, and thus the results of the hormonal treatments were calculated as relative to the NE treated group, as previously described^43^ (see Supplementary Fig. S8 for example).

### Mitochondrial DNA analysis

Frozen iBAT samples were homogenised in Qiazol (Qiagen) and incubated for 5 minutes at room temperature, after which chloroform (Sigma-Aldrich) was added. Following centrifugation, total DNA was precipitated from the interphase and the phenol phase with 100% ethanol. After further centrifugation, the resulting DNA pellet was washed in 0.1M sodium citrate, followed by 75% (v/v) ethanol. The DNA pellet was air-dried, resuspended in 8mM sodium hydroxide, and neutralised by the addition of HEPES buffer and 100 mM EDTA, and stored at −20 °C until use. Relative mitochondrial content was assessed by comparing the Ct of mitochondrial Cox2 relative to nuclear β-actin, as described above.

### Statistics

Data are presented as mean ± standard error of the mean (SEM). Statistical analysis was performed using Origin Pro software (OriginLab). An unpaired Student’s t-test was used for comparison between two groups. One-way ANOVA was used when comparing more than two groups, followed by Tukey’s post-hoc analysis. A p-value of <0.05 was considered significant. Data was assessed for normality by carrying out standard distribution plots. Where there were outliers, sensitivity analysis was performed and analysis repeated with the outlier(s) removed.

### Study Approval

All experiments were performed in accordance with the Animals (Scientific Procedures) Act 1986 Amendment Regulations 2012 and approved by King’s College London’s Animal Welfare and Ethical Review Body and the Home Office.

## Electronic supplementary material

Supplementary Information
