# Supplementary material for: A progesterone-brown fat axis is involved in regulating fetal growth
Source: Sci Rep. 2017 Sep 6;7:10671. doi: 10.1038/s41598-017-10979-7 (PMC5587669; doi:10.1038/s41598-017-10979-7)
Supplement: Supplementary file 1 — Supplementary Information [file 41598_2017_10979_MOESM1_ESM.pdf]

A progesterone-brown fat axis is involved in regulating fetal growth

Saraid McIlvride, Aleena Mushtaq, Georgia Papacleovoulou, Chloe Hurling, Jennifer Steel, Eugène Jansen, Shadi Abu-Hayyeh and Catherine Williamson

SUPPLEMENTARY INFORMATION

Supplementary Data

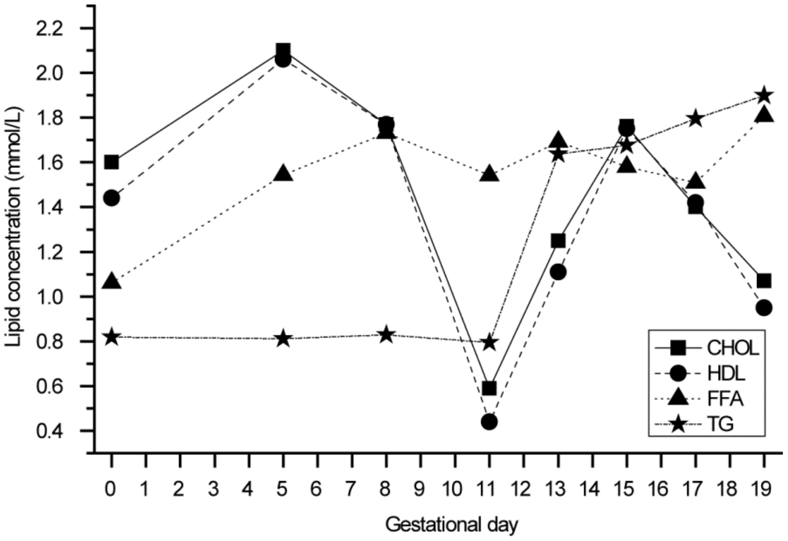

Supplementary Figure S1. Switching of serum lipid biochemistry takes place at GD11 onwards
Serum concentrations of cholesterol (CHOL), HDL-cholesterol (HDL), free fatty acids (FFA) and triglycerides (TG). Pooled sampled serum from n=11 pregnant mice.

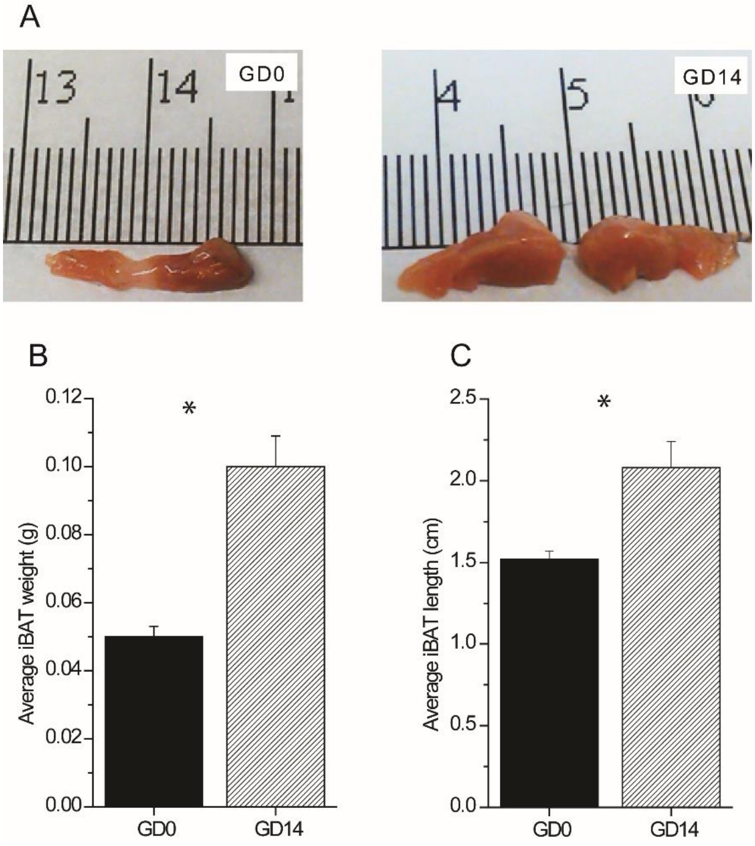

Supplementary Figure S2. Pregnancy alters the morphometry of BAT
Images of iBAT taken from non-pregnant (GD0) or day 14 pregnant (GD14) mice (A). Average iBAT weight (B) and length (C). Data represented as mean ±SEM, n=5 per group. * $p<0.05$ as determined by Student's t-test.

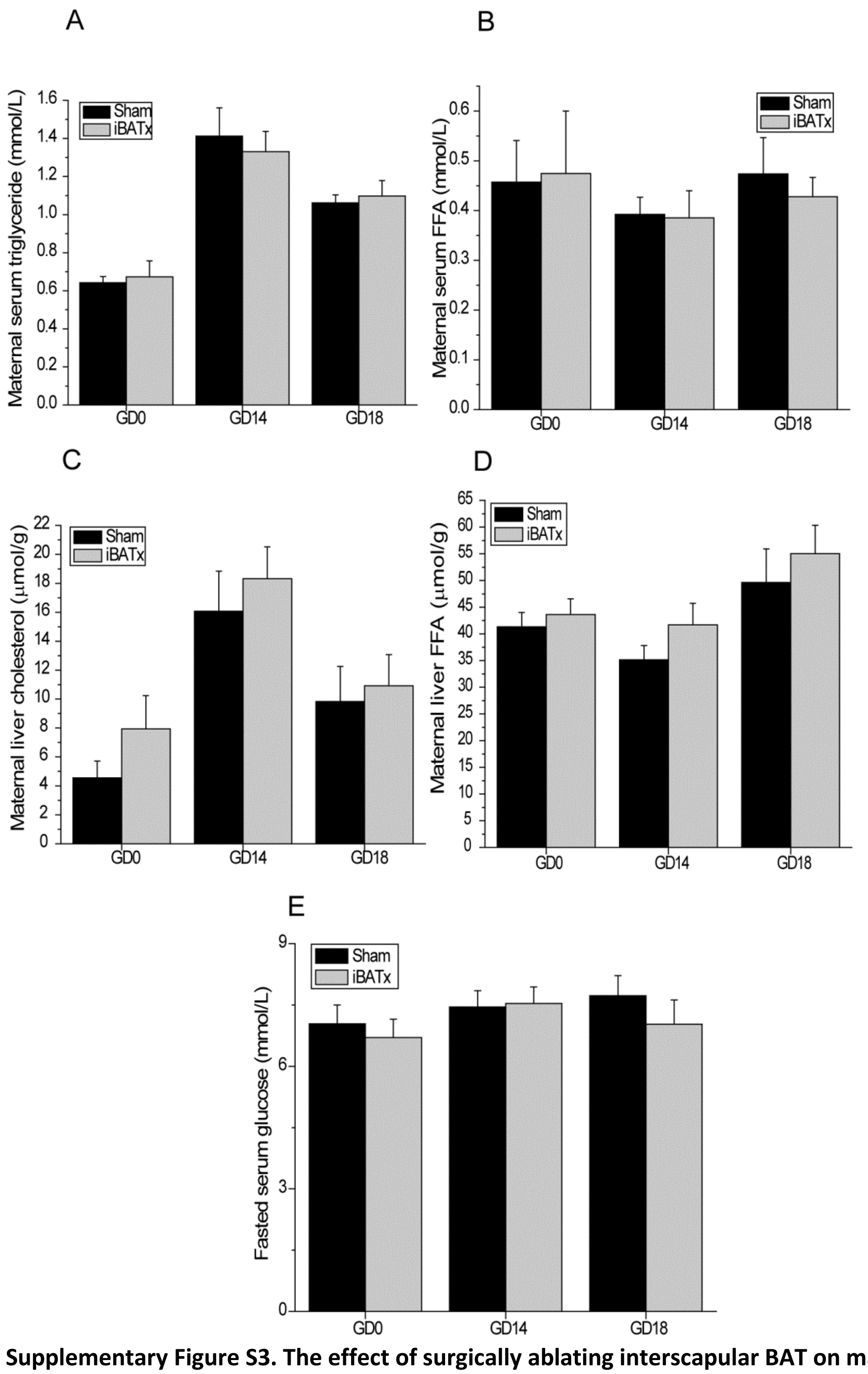

Supplementary Figure S3. The effect of surgically ablating interscapular BAT on maternal biochemistry

Lipid profiles of maternal (A) serum triglycerides, (B) serum free fatty acids (FFA), (C) liver cholesterol and (D) liver FFA. (E) Maternal fasted serum glucose. Data represented as mean ±SEM, n=6-11 per group.

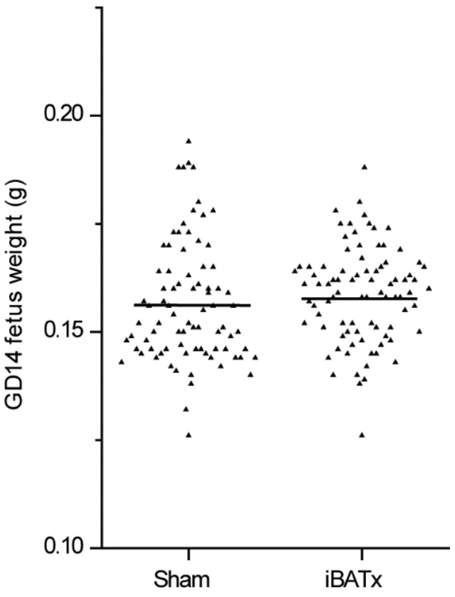

Supplementary Figure S4. Surgical ablation of maternal interscapular BAT does not alter fetal weight at gestational day 14

Sham / iBATx fetal weight: n=82 / 86 fetuses from 11 / 11 mothers.

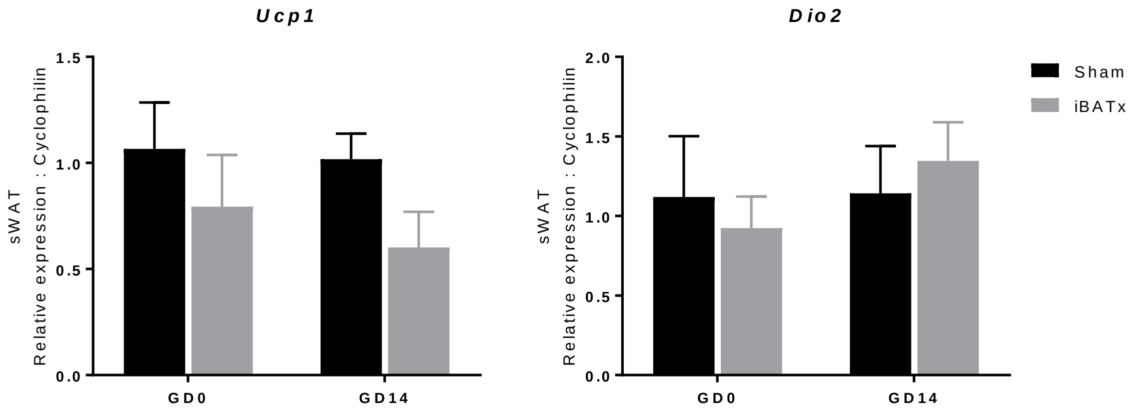

Supplementary Figure S5. Surgical ablation of maternal interscapular BAT does not alter maternal subcutaneous WAT expression of BAT marker genes *Ucp1* and *Dio2*
Data represented as mean ± SEM, n=3-6 per group.

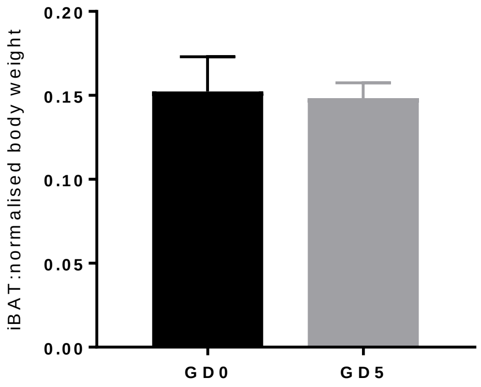

Supplementary Figure S6. No changes in normalised iBAT weight at GD5
Data represent mean ±SEM, GD0 n=4, GD5, n=7.

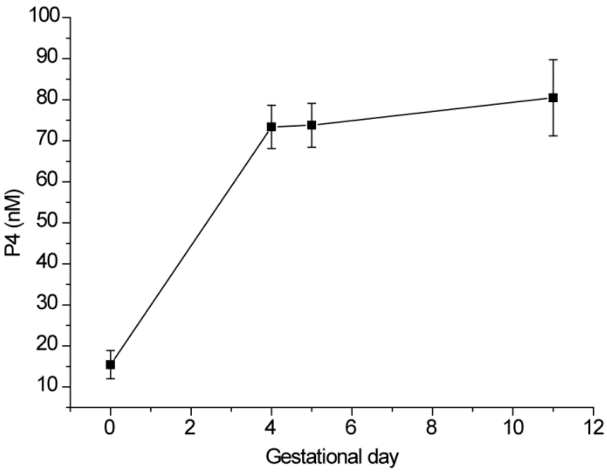

Supplementary Figure S7. Serum progesterone concentrations increase rapidly following coitus
Data represented as mean ±SEM, n=9-11.

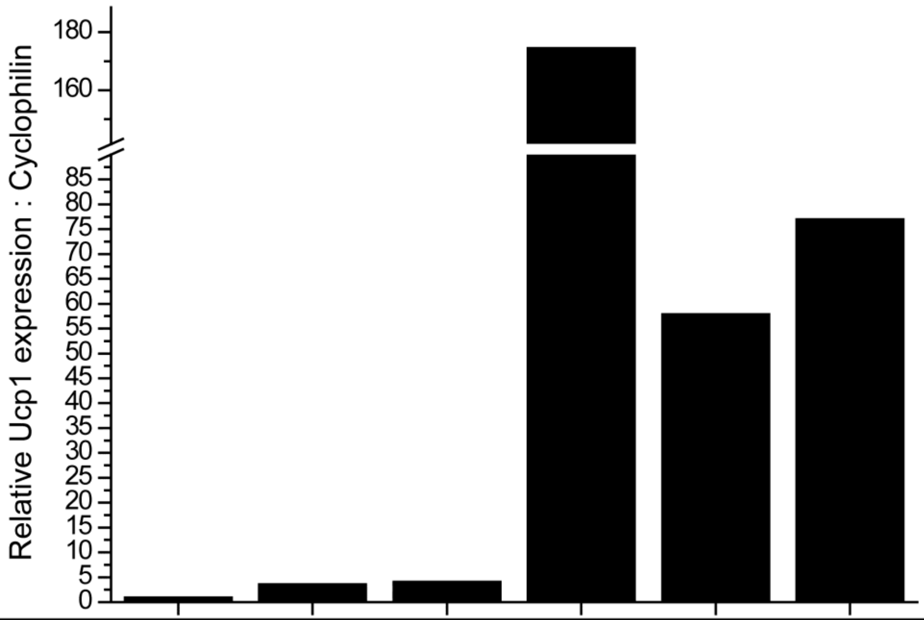

	Vehicle	P4	Prolactin	NE	NE + P4	NE + Prolactin
Ucp1 Ct	30.4	29.9	31.2	23.7	22.9	24.5
Cyclo Ct	20.0	20.7	20.6	21.3	19.1	20.8

Supplementary Figure S8. Representative example of *Ucp1* mRNA expression in cultured primary brown adipocytes +/- treatment with progesterone (P4), prolactin and norepinephrine (NE) (n=1).

Serum Progesterone Measurements. The serum progesterone concentration in mice at GD0 to GD11 was measured using the HTRF progesterone assay kit (CisBio) following a 1:15 dilution in assay buffer, according to manufacturer's instructions.

Table of Primer Sequences

Gene	Forward Primer	Reverse Primer
β-Actin	GATGACGATATCGCTGCGCTG	CTAGGGCGGCCCACGAT
Abca1	TCCTCATCCTCGTCATTCAAA	GGACTTGGTAGGACGGAACCT
Abcg1	GCTGTGCGTTTTGTGCTGTT	TGCAGCTCCAATCAGTAGTCC
Abcg5	TCAATGAGTTTTACGGCCTGAA	GCACATCGGGTGATTTAGCA
Abcg8	TGCCCACCTTCCACATGTC	ATGAAGCCGGCAGTAAGGTAG
Acc1	GGCCAGTGCTATGCTGAGAT	AGGGTCAAGTGCTGCTCCA
Acc2	ACTTTGACCTGACCGCTGTG	CTGAGTGCCGGATAATGGC
Adipoq	AGATGCAGGTCTTCTTGGTC	CTGAGCGATACACATAAGCGG
Adrb3	GGTAATCATAGCCATCGCCC	CAGTTACACAGAGCACGTCC
Adrp	TGGCAGCAGCAGTAGTGGAT	AGCTCACCAAGGGCAGGTT
Cd36	GATGTGGAACCCATAACTGGA	GGCTTGACCAATATGTTGACC
Cidea	AGAGTCACCTTCGACCTATACAG	AACCTCAGCAGATTCCTTAACAC
Cyp7a1	AGCAACTAAACAACCTGCCAGTACTA	GTCCGGATATTCAAGGATGCA
Cyclophilin B	TGGAGAGCACCAAGACAGACA	TGCCGGAGTCGACAATGAT
Dgat2	AGTGGCAATGCTATCATCATCGT	AAGGAATAAGTGGGAACCCAGATCA
Dio2	CCTACAAACAGGTTAAACTGGG	CTCTGCACTGGCAAAGTC
El	AGCGTCTATTGTTACTTCCC	TTTATGATGCTCATCTCGCAG
Fabp-pm	CAAAGATGCAGAAGAAGCC	CCTCTTGCAACCATTGCT
Fas	CCCAGAGGCTTGTGCTGACT	CGAATGTGCTTGGCTTGGT
Fatp1	CGTTTCGATGGTTATGTTAGTG	CTAGCACGTCACCTGAGAG
Fatp4	TTCCCTCATCCTCCTGCT	CGATGTTTCCTGCTGAGTG
Hmgcr	TTGGCACCATGTCAGGCGTCC	AGCGACACACAGGCCGGGAA
Hsl	CTATTCAGGGACAGAGGCAG	TAGTTCCAGGAAGGAGTTGAG
Lal	TCTTCTCAAGGACATGTTTG	CAAAGCTCCTTCATGATGAC
Lep	GTTCAAGCAGTGCCTATCC	AAGTCCAAGCCAGTGACC
Lpl	CCAGGATGCAACATTGGAGA	CAACTCAGGCAGAGCCCTTT
Lxrα	AGGAGTGTCGACTTCGCAAA	CTCTTCTTGCCGCTTCAGTT
Lxrβ	AAGCAGGTGCCAGGGTTCT	TGCATTCTGTCTCGTGGTTGT
mtCox2	TGCTCTCCCCTCTCTAC	GGTGCCCTATGGTTTTAACG
Nd5	AGCATTCGGAAGCATCTTTG	TTGTGAGGACTGGAATGCTG
Pgc1a	TGAAAGGGCCAAACAGAGAGA	TAAATCACACGGCGCTCTT
Scd1	CCCCTGCGGATCTTCCTTAT	AGGGTCGGCGTGTGTTTCT
Scd2	AGCGGGCTGCAGAAACTTAG	GGCTGAGTAAGCGCCAGAGAT
Srebp1c	GCAGCCATGGATTGCACATT	GGCCCGGGAAGTCACTGT
Ucp1	AATACTGGCAGATGACGTCC	TTACCACATCCACTGGAGAG
Zic1	CACATGAAGGTCCATGAGTCC	GGGTTGTCTGTTGTGGGAG
